# Cerebral Activity in Female Baboons (*Papio anubis*) During the Perception of Conspecific and Heterospecific Agonistic Vocalizations: a Functional Near Infrared Spectroscopy Study

**DOI:** 10.1007/s42761-022-00164-z

**Published:** 2022-11-29

**Authors:** Coralie Debracque, Thibaud Gruber, Romain Lacoste, Adrien Meguerditchian, Didier Grandjean

**Affiliations:** 1 Neuroscience of Emotion and Affective Dynamics Lab, Faculty of Psychology and Educational Sciences and Swiss Center for Affective Sciences, University of Geneva, Campus Biotech, Chemin Des Mines 9, 1202 Geneva, Switzerland; 2 Station de Primatologie-Celphedia, CNRS UARS846, Rousset-Sur-Arc, France; 3 Laboratoire de Psychologie Cognitive UMR7290, CNRS, Université Aix-Marseille, Marseille, France

**Keywords:** fNIRS, Cerebral activity, Monkey, Agonistic vocalizations, Conspecific, Heterospecific

## Abstract

The “voice areas” in the superior temporal cortex have been identified in both humans and non-human primates as selective to conspecific vocalizations only (i.e., expressed by members of our own species), suggesting its old evolutionary roots across the primate lineage. With respect to non-human primate species, it remains unclear whether the listening of vocal emotions from conspecifics leads to similar or different cerebral activations when compared to heterospecific calls (i.e., expressed by another primate species) triggered by the same emotion. Using a neuroimaging technique rarely employed in monkeys so far, functional Near Infrared Spectroscopy, the present study investigated in three lightly anesthetized female baboons (*Papio anubis*), temporal cortex activities during exposure to agonistic vocalizations from conspecifics and from other primates (chimpanzees—*Pan troglodytes*), and energy matched white noises in order to control for this low-level acoustic feature. Permutation test analyses on the extracted OxyHemoglobin signal revealed great inter-individual differences on how conspecific and heterospecific vocal stimuli were processed in baboon brains with a cortical response recorded either in the right or the left temporal cortex. No difference was found between emotional vocalizations and their energy-matched white noises. Despite the phylogenetic gap between *Homo sapiens* and African monkeys, modern humans and baboons both showed a highly heterogeneous brain process for the perception of vocal and emotional stimuli. The results of this study do not exclude that old evolutionary mechanisms for vocal emotional processing may be shared and inherited from our common ancestor.

**Supplementary Information:**

The online version contains supplementary material available at 10.1007/s42761-022-00164-z.

Since the 1990s, (George et al., 1995; Pihan et al., 1997), many neuroimaging studies have investigated the activity of the human temporal cortex during emotional voice processing. Hence, functional Magnetic Resonance Imaging (fMRI) and more recently functional Near Infrared Spectroscopy (fNIRS) data pointed out the role of the bilateral superior temporal gyrus (STG), superior temporal sulcus (STS), and middle temporal gyrus (MTG) in the processing of emotional prosody (Grandjean, 2020; Grandjean et al., 2005; Kotz et al., 2013; Plichta et al., 2011; Wildgruber et al., 2009; Zatorre & Belin, 2001) and more specifically in the recognition of positive and negative emotions (Bach et al., 2008; Frühholz & Grandjean, 2013; Johnstone et al., 2006; Zhang et al., 2018).

Despite calls for an evolutionary-based approach to emotions that considered the adaptive functions and the phylogenetic continuity of emotional expression and identification (Bryant, 2021; Greenberg, 2002), few comparative studies have investigated the human temporal cortex activity during the recognition of emotional cues in human voices (conspecific) and other species vocalizations (heterospecific), expressed especially by non-human primates (NHP), our closest relatives (Perelman et al., 2011). In the few studies published so far, fMRI conjunction analysis has interestingly identified commonalities in the human cerebral response to human and other animal vocalizations including macaques (*Macaca mulatta*) and domestic cats (*Felis catus*). More activations were indeed found in the medial posterior part of the human right orbitofrontal cortex (OFC) during the listening of agonistic vocalizations expressed by both humans and other animals compared to affiliative ones (Belin et al., 2008). On the contrary, Fritz and colleagues demonstrated a greater involvement of the human STS and the right planum temporale (PT) for the identification of human emotional voices contrasted to chimpanzees (*Pan troglodytes*) and then macaque calls (Fritz et al., 2018). Similar results were found in the STS and STG when human emotional voices were compared to various animal sounds, non-vocal stimuli, or non-biological noises (Bodin et al., 2021; Pernet et al., 2015) suggesting a sensitivity of the superior regions of the human temporal cortex but not of the frontal cortex for conspecific voices.

Is this sensitivity of the temporal cortex to emotional cues expressed by conspecifics found in NHP? In other words, are the cerebral mechanisms of vocal emotion perception shared across primate species, or has the auditory cortex of *Homo sapiens* evolved differently?

The previous literature on primates emphasizes brain continuity between humans and NHP for the auditory processing of conspecific emotions. For instance, fMRI studies in macaques have revealed a greater involvement of the STG for the perception of conspecific emotional calls compared to heterospecific ones including calls from other primate and non-primate species, environmental sounds and scrambled vocalizations (Joly et al., 2012; Ortiz-Rios et al., 2015; Petkov et al., 2008). Following this, positron emission tomography (PET scan) studies have shown the predominant role of the right PT in chimpanzees (Taglialatela et al., 2009) and of the STS in macaques (Gil-da-Costa et al., 2004) for the processing of conspecific emotional calls. Additionally, neurobiological findings in macaques and marmosets (*Callithrix jacchus*) have suggested a greater involvement of the STG and of the primary auditory cortex in the passive listening of emotional conspecific calls compared to environmental sounds, scrambled, or time-reversed vocalizations (Belin, 2006; Ghazanfar & Hauser, 2001; Poremba et al., 2004). Overall, as for humans, the literature in NHP suggests a sensitivity of the great ape and monkey temporal cortex for the processing of conspecific emotional vocalizations.

Despite these results, the question of the specific status of conspecific emotions in NHP remains poorly explored with respect to heterospecific vocalizations. In particular, because of the species-dependent results in humans highlighted above and the phylogenetic proximity across primate species, it seems necessary to include heterospecific stimuli from other NHP to reconstruct the phylogenetic evolution of primate vocal emotion processing (Bryant, 2021).

The present study investigated temporal cortex involvement in three female baboons, Talma, Rubis, and Chet, during exposure to conspecific vs. heterospecific agonistic vocalizations, using fNIRS. Building on a growing interest over the past decade (Boas et al., 2014; Pan et al., 2019), we used fNIRS because of its non-invasiveness, its poor sensitivity to motion artifacts (Balardin et al., 2017) and its suitability for comparative research (Debracque et al., 2021; Fuster et al., 2005; Kim et al., 2017; Lee et al., 2017; Wakita et al., 2010). According to the existing literature on NHP and humans suggesting a sensitivity of the primates’ temporal cortex for conspecific calls, we expected (i) more activation in the temporal cortex for the passive listening of baboon sounds compared to chimpanzee stimuli, and (ii) a greater involvement of the temporal cortex for the perception of agonistic conspecific vocalizations in comparison to the other sounds.

## Method

### Subjects

The few existing studies using fNIRS in NHP mostly include a single subject (Fuster et al., 2005; Wakita et al., 2010). Three healthy female baboons (Talma, 13.5 years old; Rubis, 18.4 years old; and Chet, 11.8 years old) were included in the present study, contingent with their yearly health check-up; this sample size was consistent with prior work on the perception of affective stimuli by female macaques (Lee et al., 2017). In addition, as male baboons have large and thick masticatory muscles above their temporal cortex, they were excluded from the experimental protocol. Sexual dimorphism being particularly pronounced in baboons (Phillips-Conroy & Jolly, 1981), the female sex was assigned to the subjects based on their facial morphology and red buttocks. Moreover, preventing any ambiguity about the subjects’ sex, the three female baboons had already given birth to offspring that they breastfed. Following this, based on the annual health assessment and the daily behavioral surveys made by the veterinary and animal welfare staff, the subjects had normal hearing abilities and did not present a structural neurological impairment (confirmed with respective T1w anatomical brain images—0.7 × 0.7 × 0.7 resolution—collected in vivo under anesthesia in the 3Tesla MRI Brunker machine). All procedures were approved by the “C2EA-71 Ethical Committee of neurosciences” (INT Marseille) and performed in accordance with the relevant French law, CNRS guidelines, and the European Union regulations. The subjects were born in captivity and housed in social groups at the Station de Primatologie in which they have free access to both outdoor and indoor areas. All enclosures are enriched by wooden and metallic climbing structures as well as substrate on the group to favor foraging behaviors. Water is available ad libitum and monkey pellets, seeds, fresh fruits, and vegetables were given every day. The three subjects were lightly anesthetized with propofol and passively exposed to auditory stimuli as described below (see also Debracque et al., 2021 for the complete protocol).

### Stimuli

**Fig. 1:**
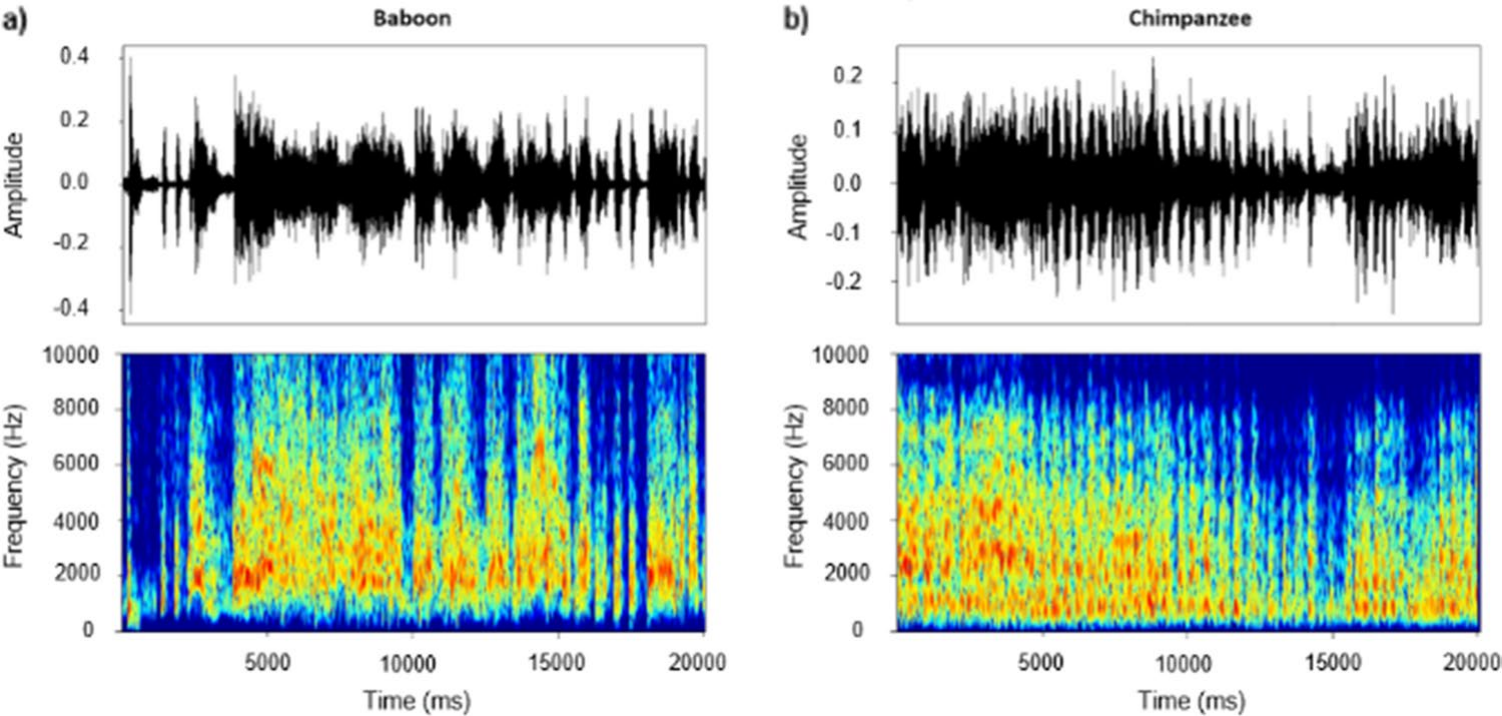
Representative waveforms and spectrograms of 20 s-long agonistic **a** baboon and **b** chimpanzee vocalizations used as stimuli in the present study. These graphical representations were extracted using the PhonTools package (Barreda, 2015) on R. studio (Team, 2020)

Auditory stimulations consisted of agonistic vocalizations produced by baboon (conspecific—see Fig. 1a) and chimpanzee (heterospecific—see Fig. 1b) individuals as well as energy-matched white noises to control for this low-level acoustic feature and for its unfolding (i.e., the temporal structure of energy of the vocalizations). Aggressor screams and distress calls expressed in an agonistic (i.e., conflictual) context are commonly used in the literature to investigate affective states associated with threat and distress respectively in primate vocalizations (Briefer, 2012; Kret et al., 2020). More specifically, studies on the baboons’ vocal repertoire have shown the link between agonistic vocalizations produced during conflicts and the threatening or distressful emotional state of the caller (Kemp et al., 2017; Seyfarth & Cheney, 2009).

Each auditory stimulus had a duration of 20 s and was repeated six times (see Debracque et al., 2021 for more details). The auditory stimuli were pseudo-randomized, alternating vocalizations, and white noises and were separated by 15 s of silence. Additionally, auditory stimulations were broadcasted either binaurally or monaurally in the right or left ear.

### fNIRS

#### Recordings

**Fig. 2:**
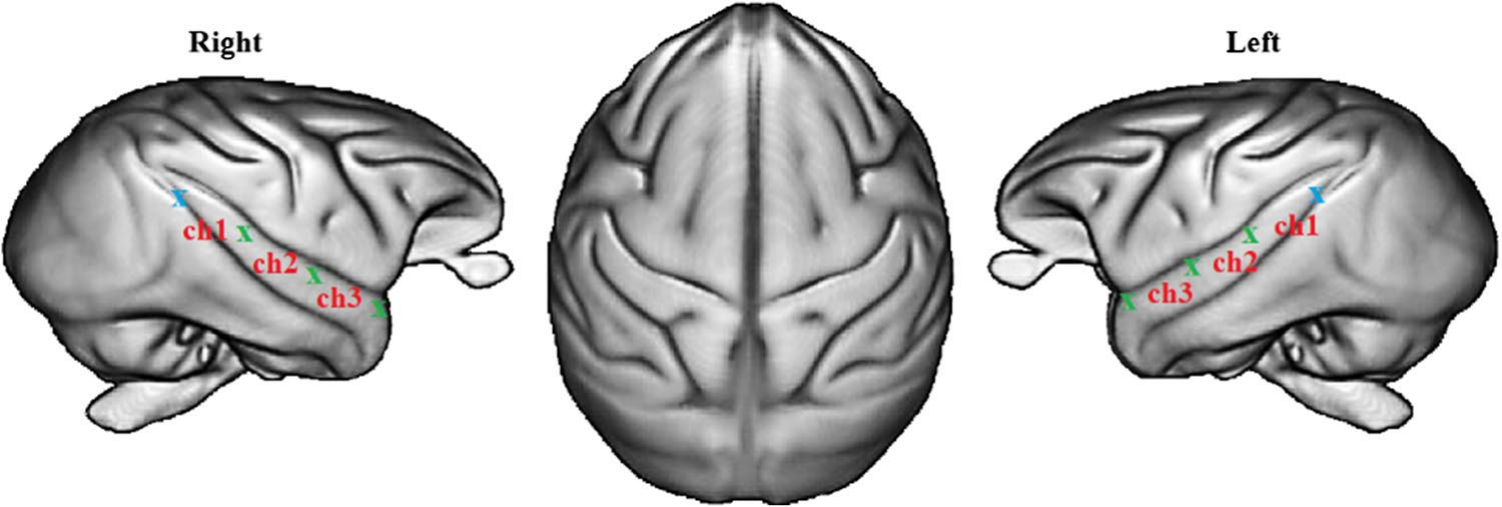
fNIRS optode and channel locations according to 89 baboons T1 MRI template (Love et al., 2016). Blue and green crosses represent optical receivers and transmitters, respectively. Ch1, Ch2, and Ch3 indicate the three channels on the right and left temporal cortex

Brain activations were measured using two light and wireless fNIRS devices (Portalite, Artinis Medical Systems B.V., Elst, The Netherlands). Based on tissue transillumination (Bright, 1831), fNIRS measures using near-infrared lights blood oxygenation changes (e.g., Hoshi, 2016; Jöbsis, 1977) related to the hemodynamic response function constituted of Oxyhemoglobin (O_2_Hb) and deoxyhemoglobin. fNIRS is a non-invasive technique poorly sensitive to motion artifacts (Balardin et al., 2017) and fully wearable. The fNIRS optical probes were placed on the right and left temporal cortices of the subjects using T1 MRI scanner images previously acquired at the Station de Primatologie on baboons (see Fig. 2). Data were obtained at 50 Hz with two wavelengths (760 and 850 nm) using three measurements, i.e., channels per hemisphere (ch1, ch2, ch3) with three inter-distance probes (3–3.5–4 cm) investigating three different cortical depths (1.5–1.7–2 cm, respectively).

Reducing the potential disturbing impact of the fNIRS protocol on the subjects, each experimental session was planned during the baboons’ routine health inspection at the Station de Primatologie. As part of the health check, subjects were isolated from their social group and anesthetized with an intramuscular injection of ketamine (5 mg/kg—Ketamine 1000®) and medetomidine (50 µg/kg—Domitor®). Then Sevoflurane (Sevotek®) at 3 to 5% and atipamezole (250 µg/kg—Antisedan®) were administered before recordings. Each baboon was placed in ventral decubitus position on the table, and the head of the individual was maintained using foam positioners, cushions, and Velcro strips to remain straight and to reduce potential motion occurrences. Vital functions were monitored (SpO2, Respiratory rate, ECG, EtCO2, T°), and a drip of NaCl was put in place during the entire anesthesia. Before fNIRS recordings, temporal areas on the baboons’ scalps were shaved and sevoflurane inhalation was stopped. Subjects were further sedated with a minimal amount of intravenous injection of Propofol (Propovet®) with a bolus of around 2 mg/kg every 10 to 15 min or by infusion rate of 0.1–0.4 mg/kg/min. After the recovery period, subjects were put back in their social group and monitored by the veterinary staff.

#### Analysis

SPM_fNIRS toolbox (Tak et al., 2016) and custom-made codes on Matlab 7.4 R2009b (The MathWorks Inc., 2009) were used to perform first-level analysis on raw fNIRS data following this procedure: (i) O_2_Hb concentration changes were calculated using the Beer-Lambert law (Delpy et al., 1988); (ii) motion artifacts were removed manually in each individual and each channel. In total, 10 s (1.3%) were removed from the O_2_Hb signal of Rubis and 35 s (4.8%) for Talma and Chet; (iii) a low-pass filter based on the HRF (Friston et al., 2000) was applied to reduce physiological confounds; (iv) a baseline correction was applied in subtracting the pre-stimulus baseline from the post-stimulus O_2_Hb concentration changes of each trial and (v) O_2_Hb signal was averaged for Talma in a window of 4 to 12 s post-stimulus onset for each trial and for Rubis and Chet in a window of 2 to 8 s post-stimulus onset in order to select the range of maximum O_2_Hb concentration changes following Debracque et al. (2021). Shortly, the differences of concentration range are explained by the presence of tachycardia episodes for both Rubis and Chet during the experiment, involving an HRF almost twice as fast as the one found for Talma.

The second level analysis was made on R. studio (Team, 2020) using the permuco package (Frossard & Renaud, 2019). Through the same data sample, we already demonstrated in Debracque et al.(2021) the robustness of our method and results regarding hemispheric lateralization following motor and auditory stimulations. In the present paper, we wanted to investigate a higher level of the brain process, i.e., the perception of conspecific and heterospecific sounds. Hence, in each *Hemisphere* (right, left), we used non-parametric permutation tests with 5,000 iterations to assess O_2_Hb concentration changes for each subject (Talma, Rubis, Chet) as they enable repeated measures ANOVA in small sample sizes by multiplying the design and response variables (Kherad-Pajouh & Renaud, 2015). *Stimuli* (call, white noise); *Species* (baboon, chimpanzee); *Channels* (ch1, ch2, ch3), and *Stimulus sides* (right, left, both ears) were selected as fixed factors. As recommended, contrast effects of *Species* and *Stimuli* within channels were assessed with 2,000 permutations (Kherad-Pajouh & Renaud, 2015). Both *p* values for permutation *F* (p_perm_) and parametric *F* are reported.

## Results

**Fig. 3:**
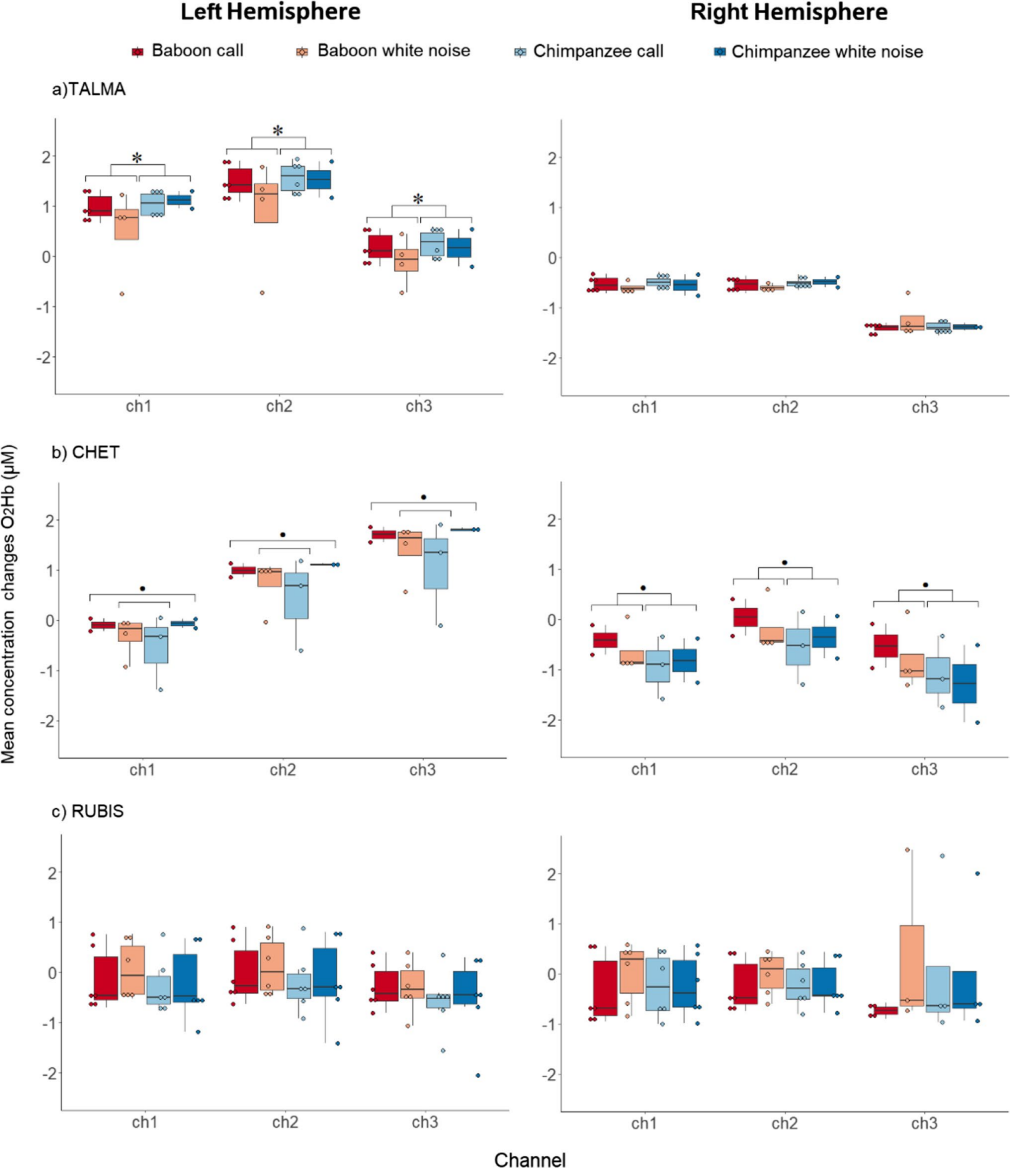
Right and left temporal cortex activations for the baboons **a** Talma, **b** Chet, and **c** Rubis during the perception of agonistic baboon (conspecific) and chimpanzee (heterospecific) vocalizations as well as their energy-matched white noises. The mean concentration changes of O_2_Hb (*y* axis) are represented in micromolar (µM) for each fNIRS channel (*x* axis). Colorful dots and dark lines represent stimuli and confidence intervals respectively. Results of the permutation tests within channels are shown with *p < 0.05; *p* = 0.07. The ggplot2 package (Wickham et al., 2021) on R.studio (Team, 2020) was used for visualizing the data

First, regarding the subject Talma, permutation tests revealed significant differences of O_2_Hb concentration changes between the three *Channels* for the right, *F* (2,3) = 161.5, *p* | p_perm_ < 0.001 and left hemisphere, *F* (2,3) = 33.91, *p* | p_perm_ < 0.001. The main factor *Species* was also found significant for the left hemisphere only, right: *F* (1,2) = 0.34, *p* | p_perm_ = 0.057; left: *F* (1,2) = 4.24, *p* | p_perm_ < 0.05. The main factor *Stimuli* as well as the interactions Stimuli*Species, Stimuli*Channels, Species*Channels, and Stimuli*Species*Channels did not reach significance within the right or left hemisphere (see Supplementary Material). Following these analyses, contrasts within each channel showed that for Talma’s left hemisphere, the perception of baboon sounds led to lower O_2_Hb concentration changes compared to chimpanzee sounds in ch1, ch2, and ch3, right: *F* (1,2) = 0.15, *p* | p_perm_ = 0.69; left: *F* (1,2) = 4.07, *p* | p_perm_ = 0.05—see Fig. 3a.

Second, for the subject Chet, significant differences of O_2_Hb concentration changes between the three *Channels* were found for the right, right: *F* (2,3) = 3,99, *p* | p_perm_ < 0.05 and left hemisphere, *F* (2,3) = 25.68, *p* | p_perm_ < 0.001. The main factor *Species* was also found significant for the right hemisphere only, right: *F* (1,2) = 5.03, *p* | p_perm_ < 0.05; left: *F* (1,2) = 0.24, *p* | p_perm_ = 0.62. Additionally, statistics showed a significant two-way interaction between *Stimuli* and *Species* for the left hemisphere only, right: *F* (1,2) = 0.01, *p* | p_perm_ = 1; left: *F* (1,2) = 4.13, *p* | p_perm_ = 0.05. The main factor *Stimuli* as well as the interactions Stimuli*Channels, Species*Channels and Stimuli*Species*Channels did not reach significance within the right or left hemisphere (see Supplementary Material). Finally, contrasts within each channel revealed that for Chet, while her right hemisphere had a tendency to be more activated for the perception of baboon sounds compared to chimpanzee stimuli in ch1, ch2, and ch3, right: *F* (1,2) = 3.74, *p* | p_perm_ = 0.07; left: *F* (1,2) = 0.22, *p* | p_perm_ = 0.65, her left hemisphere had a tendency to be more activated for the passive listening of baboon agonistic calls and chimpanzee white noises when compared to baboon white noises and chimpanzee agonistic calls in ch1, ch2, and ch3, right: *F* (1,2) = 0.01, *p* | p_perm_ = 1; left: *F* (1,2) = 3.75, *p* | p_perm_ = 0.07—see Fig. 3b.

Third, for the subject Rubis, only a significant difference of O_2_Hb concentration changes between the three *Channels* was found for the right hemisphere, right: *F* (2,3) = 8, 99, *p* | p_perm_ < 0.001; left: *F* (2,3) = 2.15, *p* | p_perm_ = 0.12—see Fig. 3c. None of the other main effects or interactions reached significance within the left or right hemisphere (see Supplementary Material).

Note that for Talma, Rubis, and Chet, the factor *Stimulus sides* (sounds broadcasted either binaurally or monaurally in the right or left ear) did not reach significance and thus, do not statistically explain differences in O_2_Hb concentration changes underpinning the perception of baboon and chimpanzee sounds by our three subjects (see Debracque et al., 2021 for more details related to auditory asymmetries).

In sum, across the three channels, more O_2_Hb concentration changes were found in Talma’s left temporal cortex for the perception of chimpanzee sounds compared to baboon stimuli. Conversely, for Chet, permutation analyses revealed more O_2_Hb concentration changes in the right temporal cortex for the passive listening of baboon sounds, especially baboon agonistic calls comparing to chimpanzee stimuli. Additionally, her left temporal cortex was more activated by baboon agonistic calls and chimpanzee white noises than for chimpanzee agonistic calls and baboon white noises. For Rubis, the perception of baboon and chimpanzee sounds did not affect the O_2_Hb concentration changes of her bilateral temporal cortex. Finally, to the exception of Rubis’ left hemisphere, the different cortical depths of the channels (1.5–1.7–2 cm) had an impact on the O_2_Hb measurement of Talma, Chet, and Rubis’ temporal cortices.

## Discussion

The present fNIRS study in baboons underlines a highly heterogeneous process for the auditory perception of conspecific and heterospecific affective stimuli.

Using valid statistical methods and analyses (Debracque et al., 2021; Lee et al., 2017) as well as the inclusion of three subjects instead of one, as is usually the case in the relevant literature (Fuster et al., 2005; Wakita et al., 2010), fNIRS data revealed large inter-individual differences between Talma and Chet for the significant main effect *Species*. The left temporal cortex of Talma was overall more activated for chimpanzee sounds (calls and white noise) than for baboon ones; in contrast, results were reversed in the right temporal cortex of Chet, where statistical analyses highlighted an increase of O_2_Hb concentration changes for the passive listening of agonistic baboon sounds, especially baboon agonistic calls compared to chimpanzee sounds. In addition, in her left temporal cortex, we documented an increase in O_2_Hb concentration led by both the perception of agonistic baboon calls and chimpanzee white noises. No significant results were found for Rubis, although this may have been a consequence of her constant tachycardia during the health check and experiment (see Debracque et al., 2021).

Beyond this apparent absence of congruence in our fNIRS data, our results underlined in fact a highly heterogeneous process for auditory perception in baboons. Well-known in neuroscience research with human participants, inter-individual differences are for instance at play in the location of voice-selective areas in the human auditory cortex (Belin et al., 2000). In the same line, Pernet and colleagues also demonstrated using fMRI, a great inter-individual variability in the involvement of human STG and STS for the listening of conspecific emotional voices compared to non-vocal sounds (Pernet et al., 2015). As for humans, the location of the voice-selective areas as well as the cortical response of the superior temporal cortex in baboons would be subject to a high heterogeneity. This claim is in line with the results of Xu et al. (2019) who have shown in five anesthetized and awake macaques great inter-individual variabilities in the functional connectivity of different cortical regions. Interestingly, the authors compared macaques’ fMRI data to human ones and concluded on a similar heterogeneity in functional connectivity across primate species. As highly cited human neuroimaging studies (Szucs & Ioannidis, 2020), future non-invasive comparative research should include more subjects to take in consideration this inter-individual variability in brain mechanisms. The necessity to increase NHP subjects to address limits in statistical power faces ethical aspects related to animal welfare in the case of invasive neuroimaging studies. The development of fNIRS (Debracque et al., 2021) and longitudinal studies in comparative neuroscience (Song et al., 2021) would thus allow answering parts of these challenges.

Often explored using fMRI or Pet scan (e.g., Bodin et al., 2021; Gil-da-Costa et al., 2004; Ortiz-Rios et al., 2015), our fNIRS data remain however inconclusive regarding the processing of conspecific vocalizations compared to white noises. In contrast, comparative research on macaques and marmoset (*Callithrix jacchus*) showed a greater sensitivity of the temporal cortex in its anterior part than in its posterior area for the contrast conspecific calls vs. control sounds (Bodin et al., 2021). Future fNIRS studies would help determinate whether this lack of effect replication from our present study might be addressed by improving the probe location on the scalp of baboons and its spatial sensitiveness to this expected effect.

Finally, out of the scope of this paper, permutation test analyses highlighted consistent fNIRS data for the channels 1 and 2 compared to channel 3 on both the right and left hemispheres. This result suggests that, for fNIRS in baboons, the best inter-probe distance to assess cortical activations in the temporal cortex would be between 3 and 3.5 cm. Interestingly, these distances are commonly used for fNIRS experiments in human adults (Ferrari & Quaresima, 2012).

To conclude, our fNIRS data mainly pointed out the existence of a highly heterogonous process across individuals for the perception of conspecific emotional vocalizations in baboons. Whereas such an inter-individual heterogeneity is also well documented in humans, we do thus not exclude a potential phylogenetic continuity with non-human primates in the brain processing of conspecific emotional vocalizations which might be inherited from our common ancestor. Our results remain however inconclusive, notably in regards to the lack of contrasts in conspecific agonistic vocalizations vs. white noises (control sounds), which are often explored meaningfully using fMRI or Pet scan (e.g., Bodin et al., 2021; Gil-da-Costa et al., 2004; Ortiz-Rios et al., 2015). This highlights one of the limitations of our study. fNIRS, Pet scan, and fMRI measure hemodynamic changes, however, the latter have a much higher spatial resolution (Gosseries et al., 2008) than fNIRS (Scholkmann et al., 2014). Secondly, another limitation is that our experiment focused on baboons and it is unclear whether it will replicate in other NHP species such as Central and South American monkeys. In fact, Fitch and Braccini (2013) have already suggested differences between monkeys in terms of mechanisms for the processing of conspecific and heterospecific vocalizations. A final limitation of our study is that only agonistic vocalizations were included in the present fNIRS protocol. Similarly to humans, NHP might have some distinctive brain mechanisms for negative and positive emotions (e.g., Davidson, 1992; Frühholz & Grandjean, 2013; Zhang et al., 2018).

Overall, our study does not exclude the existence of common evolutionary roots for auditory processing across primate species to explain the inter-individual variability generally reported in those studies and underlines the importance of comparative research in monkeys to understand brain mechanisms at play in modern humans.

## Supplementary Information

Below is the link to the electronic supplementary material.

Supplementary file1 (DOCX 20 KB)

## Additional Information

### Data Availability

Raw data are freely available 10.26037/yareta:3h7qyho5p5dmtmwgqstii3apgy.

### Code Availability

Custom Matlab and R. studio codes are https://yareta.unige.ch/deposit/73c79ecc-89ac-46e8-8022-54fa3f0c3cb7/detail/d9ff0c1d-dd7f-46c9-b2c6-84a6846c0f36/files.
