# Supplementary material for: Cerebral Activity in Female Baboons (Papio anubis) During the Perception of Conspecific and Heterospecific Agonistic Vocalizations: a Functional Near Infrared Spectroscopy Study
Source: Affect Sci. 2022 Nov 29;3(4):783–91. doi: 10.1007/s42761-022-00164-z (PMC9743891; doi:10.1007/s42761-022-00164-z)
Supplement: Supplementary file 1 — Supplementary file1 (DOCX 20 KB) [file 42761_2022_164_MOESM1_ESM.docx]

**Supplementary Material**

**Table S1:** Results from permutation tests with 5000 permutations for each hemisphere and subject. Abbreviations: (RH) right hemisphere; (LH) left hemisphere. Significant analyses are highlighted in light green.

|  | **Talma** | | **Rubis** | | **Chet** | |
| --- | --- | --- | --- | --- | --- | --- |
|  | **RH** | **LH** | **RH** | **LH** | **RH** | **LH** |
| ***Main effect*** |  | | | | | |
| *Species* | *F*(1,2)=0.34  p\|p_perm_=.57 | *F*(1,2)=4.24  p\|p_perm_<.05 | *F*(1,2)=0.13  p\|p_perm_=.71 | *F*(1,2)=2.59  p\|p_perm_=.12 | *F*(1,2)=5.03  p\|p_perm_<.05 | *F*(1,2)=0.24  p\|p_perm_=.62 |
| *Stimuli* | *F*(1,2)=0.04  p\|p_perm_=.95 | *F*(1,2)=1.87  p\|p_perm_=.18 | *F*(1,2)=1.21  p\|p_perm_=.27 | *F*(1,2)=0.25  p\|p_perm_=.62 | *F*(1,2)=0.13  p\|p_perm_=.72 | *F*(1,2)=0.79  p\|p_perm_=.38 |
| *Channels* | *F*(2,3)=161  p\|p_perm_<.001 | *F*(2,3)=33.9  p\|p_perm_<.001 | *F*(2,3)=8.99  p\|p_perm_<.001 | *F*(2,3)=2.15  p\|p_perm_=.12 | *F*(2,3)=3.99  p\|p_perm_<.05 | *F*(2,3)=25.7  p\|p_perm_<.001 |
| *Stimulus sides* | *F*(2,4)=0.03  p\|p_perm_=.96 | *F*(2,4)=1.59  p\|p_perm_=.21 | *F*(2,4)=0.8  p\|p_perm_=.45 | *F*(2,4)=0.13  p\|p_perm_=.87 | *F*(2,4)=1.49  p\|p_perm_=.25 | *F*(2,4)=0.71  p\|p_perm_=.51 |
| ***Interaction*** |  | | | | | |
| *Stimuli **  *Species* | *F*(1,2)=1.15  p\|p_perm_=.74 | *F*(1,2)=2.23  p\|p_perm_=.15 | *F*(1,2)=1.25  p\|p_perm_=.27 | *F*(1,2)=0.05  p\|p_perm_=.82 | *F*(1,2)=0.01  p\|p_perm_=1 | *F*(1,2)=4.13  p\|p_perm_=.05 |
| *Stimuli **  *Channels* | *F*(2,3)=1.3  p\|p_perm_=.29 | *F*(2,3)=0.17  p\|p_perm_=.85 | *F*(2,3)=0.26  p\|p_perm_=.77 | *F*(2,3)=0.03  p\|p_perm_=.96 | *F*(2,3)=0.09  p\|p_perm_=.92 | *F*(2,3)=0.02  p\|p_perm_=.98 |
| *Species **  *Channels* | *F*(2,3)=0.35  p\|p_perm_=.72 | *F*(2,3)=0.07  p\|p_perm_=.94 | *F*(2,3)=0.01  p\|p_perm_=1 | *F*(2,3)=0.01  p\|p_perm_=.99 | *F*(2,3)=0.06  p\|p_perm_=.96 | *F*(2,3)=0.01  p\|p_perm_=.99 |
| *Stimuli **  *Species **  *Channels* | *F*(2,3)=0.65  p\|p_perm_=.53 | *F*(2,3)=0.18  p\|p_perm_=.83 | *F*(2,3)=0.25  p\|p_perm_=.78 | *F*(2,3)=0.04  p\|p_perm_=.96 | *F*(2,3)=0.05  p\|p_perm_=.95 | *F*(2,3)=0.05  p\|p_perm_=.95 |

**Table S2:** Results from permutation tests with 2000 permutations for each channel and subject in left hemisphere. Abbreviations: (ch1) channel 1; (ch2) channel 2; (ch3) channel 3. Analyses toward significance are highlighted in light green.

|  | **Talma** | | | **Rubis** | | | **Chet** | | |
| --- | --- | --- | --- | --- | --- | --- | --- | --- | --- |
|  | **ch1** | **ch2** | **ch3** | **ch1** | **ch2** | **ch3** | **ch1** | **ch2** | **ch3** |
| ***Main effect*** |  | | | | | | | | |
| *Species* | *F*(1,2)=4.07  p\|p_perm_=.05 | *F*(1,2)=4.07  p =.05  p_perm_=.04 | *F*(1,2)=4.07  p\|p_perm_=.05 | *F*(1,2)=2.32  p\|p_perm_=.13 | *F*(1,2)=2.32  p\|p_perm_=.13 | *F*(1,2)=2.32  p\|p_perm_=.13 | *F*(1,2)=0.22  p =.65  p_perm_=.63 | *F*(1,2)=0.22  p =.65  p_perm_=.64 | *F*(1,2)=0.22  p\|p_perm_=.65 |
| *Stimuli* | *F*(1,2)=1.8  p\|p_perm_=.19 | *F*(1,2)=1.8  p\|p_perm_=.18 | *F*(1,2)=1.8  p\|p_perm_=.19 | *F*(1,2)=0.22  p =.64  p_perm_=.65 | *F*(1,2)=0.22  p\|p_perm_=.64 | *F*(1,2)=0.22  p\|p_perm_=.64 | *F*(1,2)=0.69  p\|p_perm_=.41 | *F*(1,2)=0.69  p =.41  p_perm_=.4 | *F*(1,2)=0.69  p =.41  p_perm_=.42 |
| *Stimulus sides* | *F*(2,4)=1.4  p\|p_perm_=.26 | *F*(2,4)=1.4  p\|p_perm_=.26 | *F*(2,4)=1.4  p\|p_perm_=.26 | *F*(2,4)=0.12  p =.89  p_perm_=.88 | *F*(2,4)=0.12  p =.89  p_perm_=.88 | *F*(2,4)=0.12  p\|p_perm_=.89 | *F*(2,4)=0.42  p\|p_perm_=.66 | *F*(2,4)=0.42  p =.66  p_perm_=.65 | *F*(2,4)=0.42  p =.66  p_perm_=.65 |
| ***Interaction*** |  | | | | | | | | |
| *Species**  *Stimuli* | *F*(1,2)=2.15  p =.15  p_perm_=.16 | *F*(1,2)=2.15  p\|p_perm_=.15 | *F*(1,2)=2.15  p =.15  p_perm_=.16 | *F*(1,2)=0.05  p\|p_perm_=.82 | *F*(1,2)=0.05  p\|p_perm_=.82 | *F*(1,2)=0.05  p\|p_perm_=.82 | *F*(1,2)=3.75  p =.07  p_perm_=.06 | *F*(1,2)=3.75  p\|p_perm_=.07 | *F*(1,2)=3.75  p =.07  p_perm_=.06 |

**Table S3:** Results from permutation tests with 2000 permutations for each channel and subject in right hemisphere. Abbreviations: (ch1) channel 1; (ch2) channel 2; (ch3) channel 3. Analyses toward significance are highlighted in light green.

|  | **Talma** | | | **Rubis** | | | **Chet** | | |
| --- | --- | --- | --- | --- | --- | --- | --- | --- | --- |
|  | **ch1** | **ch2** | **ch3** | **ch1** | **ch2** | **ch3** | **ch1** | **ch2** | **ch3** |
| ***Main effect*** |  | | | | | | | | |
| *Species* | *F*(1,2)=0.15  p\|p_perm_=.70 | *F*(1,2)=0.15  p\|p_perm_=.69 | *F*(1,2)=0.15  p\|p_perm_=.69 | *F*(1,2)=0.12  p\|p_perm_=.72 | *F*(1,2)=0.12  p =.72  p_perm_=.73 | *F*(1,2)=0.12  p\|p_perm_=.72 | *F*(1,2)=3.74  p\|p_perm_=.07 | *F*(1,2)=3.74  p =.07  p_perm_=.06 | *F*(1,2)=3.74  p =.07  p_perm_=.06 |
| *Stimuli* | *F*(1,2)=0.03  p =.98  p_perm_=.99 | *F*(1,2)=0.03  p =.98  p_perm_=.99 | *F*(1,2)=0.03  p =.98  p_perm_=.99 | *F*(1,2)=1.12  p =.29  p_perm_=.3 | *F*(1,2)=1.12  p\|p_perm_=.29 | *F*(1,2)=1.12  p =.29  p_perm_=.28 | *F*(1,2)=0.09  p =.76  p_perm_=.75 | *F*(1,2)=0.09  p\|p_perm_=.76 | *F*(1,2)=0.09  p\|p_perm_=.76 |
| *Stimulus sides* | *F*(2,4)=0.04  p\|p_perm_=.96 | *F*(2,4)=0.04  p\|p_perm_=.96 | *F*(2,4)=0.04  p\|p_perm_=.96 | *F*(2,4)=0.75  p =.48  p_perm_=.47 | *F*(2,4)=0.75  p =.48  p_perm_=.49 | *F*(2,4)=0.75  p =.48  p_perm_=.47 | *F*(2,4)=0.91  p =.42  p_perm_=.4 | *F*(2,4)=0.91  p =.42  p_perm_=.43 | *F*(2,4)=0.91  p\|p_perm_=.42 |
| ***Interaction*** |  | | | | | | | | |
| *Species**  *Stimuli* | *F*(1,2)=0.11  p\|p_perm_=.74 | *F*(1,2)=0.11  p =.74  p_perm_=.73 | *F*(1,2)=0.11  p =.74  p_perm_=.75 | *F*(1,2)=1.22  p =.27  p_perm_=.28 | *F*(1,2)=1.22  p\|p_perm_=.27 | *F*(1,2)=1.22  p\|p_perm_=.27 | *F*(1,2)=0.01  p\|p_perm_=1 | *F*(1,2)=0.01  p\|p_perm_=1 | *F*(1,2)=0.01  p\|p_perm_=1 |
